# Renal damage after liver transplantation

**DOI:** 10.1042/BSR20191187

**Published:** 2020-01-06

**Authors:** Thorsten Feldkamp, Anja Bienholz, Andreas Paul, Fuat H. Saner

**Affiliations:** 1 Department of Nephrology, University Hospital Essen, University Duisburg-Essen, Essen, Germany; 2 Department of Nephrology and Hypertension, University Hospital Schleswig-Holstein, Christian Albrechts University, Kiel, Germany; 3 Department of General-, Visceral- and Transplant Surgery, University Hospital Essen, University Duisburg-Essen, Essen, Germany

**Keywords:** acute kidney injury, cirrhosis, renal replacement, Transplantation

## Abstract

Background: Patients following liver transplantation are at risk to develop acute kidney injury (AKI). The aim of our study was to assess risk factors for the development of AKI and the impact of AKI on the outcome of patients after liver transplantation (OLT).

Patients and methods: In this retrospective study, we analyzed 149 patients undergoing OLT from 1/2004 to 12/2007. AKI was defined according to the KDIGO definition representing the AKIN and the RIFLE classification, and according to the need for renal replacement therapy (RRT).

Results: According to the AKIN criteria alone 14 patients, according to the RIFLE criteria alone no patient and according to both definitions 30 patients developed AKI. RRT was required in 54 patients experiencing AKI, whereas 51 patients did not develop AKI. Pre OLT serum creatinine (SCr) significantly predicted the development of AKI requiring RRT, but not AKI without RRT requirement. Survival rate was significantly inferior after 28 days, one or three years in patients with AKI requiring RRT (70.4, 46.4, 44.4% vs. 100, 92.2, 90.2%, *P* < 0.001). There was no difference in survival between patients experiencing AKI according to the RIFLE or AKIN criteria without RRT requirement and patients without AKI.

Conclusion: Pre OLT renal dysfunction assessed by SCr was the most important risk factor predicting severe forms of AKI, but not milder forms of AKI. AKI requiring RRT had a detrimental impact on patients’ survival, whereas milder forms of AKI were not associated with a worse outcome.

## Introduction

Patients following orthotopic liver transplantation (OLT) are at major risk to develop acute kidney injury (AKI) [1–4]. Factors that may contribute to postoperative AKI may be preoperative hepatorenal syndrome, extended cava cross clamping time, perioperative hypotension and massive transfusion [3]. Immunosuppression protocols with delayed use of calcineurin-inhibitor (CNI) seem to be protective against AKI and also prevent chronic renal damage [5]. AKI has been proposed to be a risk factor for morbidity and mortality [6]. The development of AKI has tremendous impact on short-term outcome but also on long-term outcome up to 10 years after the primary insult [7–9].

There is an ongoing discussion about the definition of AKI [7,10], which resulted in more than 35 different definitions of AKI used in different studies and published in the literature [7]. This heterogeneity of definitions limited comparisons of risk factors and outcomes across study populations. To overcome these flaws, in 2004 the Acute Dialysis Qualitative Initiative (ADQI) group [11] established a general definition of AKI, called the RIFLE criteria. On the basis of glomerula filtration rate, serum-creatinine (SCr), and urine output the ADQI group defined five categories for renal damage. This definition was refined by the Acute Kidney Injury Network (AKIN) group [8] focusing on immediate changes in serum creatinine and urine output. The AKIN group also coined the term acute kidney injury, implicating that not only complete renal failure has an impact on patient’s outcome, but also milder renal damage would have an impact on the outcome of the patient. The major difference of the definition of the AKIN group compared with RIFLE was that modest changes in SCr (>0.3 mg/dl) were already considered as AKI. This was done because of the accumulating studies, showing that these modest changes of SCr had a significant impact on mortality [12–14]. Both definitions have now been extensively validated in different cohorts, showing good discrimination in outcome parameters between the different stages of AKI. Currently, the kidney disease improving global outcome (KDIGO) group revised the RIFLE and AKIN definition [15]. The definition of AKI proposed by the KDIGO group is a synthesis of both definitions. In the present study, we evaluate the impact of AKI, defined by KDIGO representing the RIFLE and the AKIN definition, and the impact of AKI requiring renal replacement therapy (RRT) in patients after liver transplantation. Additionally, we study the risk factors for AKI in this patient cohort.

## Patients and methods

This retrospective, single-center, cohort study was approved by the local ethics committee and followed the Declaration of Helsinki. The ethics committee waived informed consent due to retrospective design. All consecutive liver transplant patients between 1/2004 and 12/2007, who were admitted to the surgical intensive care unit (ICU), with and without AKI, were included into the analysis. None of the patients required dialysis treatment before transplantation. Exclusion criteria were pediatric patients (age <18 years) and incomplete patients records. All patients were transplanted using a unique surgical technique with Picky back, without portal-caval shunting. Transplantation was done classical with cava replacement, and a strict anesthetic protocol was followed. Postoperatively, all patients were treated at a single intensive care unit (ICU) with standardized ICU treatment applying standardized care consisting of triple immune suppression (corticosteroids, mycophenolat mofetile and tacrolimus or cyclosporine A) [16]. CRRT was initiated during the course of AKI [8,11], if potassium levels increased to ≥ 5.5 mmol/l or patients were significantly volume overloaded. RRT was performed primarily as continuous veno-venous hemodialysis (CVVHD) as previously described [4]. Liver function tests, electrolytes, creatinine, blood urea nitrogen and total calcium were measured at least once daily.

### Variables

Several parameters were examined for an association with AKI: Pre and intra OLT factors such as gender, age, height, weight, body mass index (BMI), SCr, bilirubin, INR, cold ischemia time, warm ischemia time and Model End-Stage liver Disease (MELD) score were investigated for association with AKI. MELD score was determined for each patient using the following equation: 10 × [0.967log_e_ (SCr [mg/dl]) + 0.38log_e_ (bilirubin [mg/dl]) + 1.12 log_e_ (INR) + 0.643] [17]. Indications for OLT were recorded. Post OLT complications, within the hospital stay, such as time on respirator and transfusion of red packed cells (RPC) were evaluated. The patient outcomes of days on the ICU and short term (28 days) and long-term (one year and three year) survival were calculated. Survival was determined by calculating the time from OLT-date until death or end of observational period. Finally, the influence of pre and intra OLT factors on patient survival were evaluated including gender, age, height, weight, body mass index (BMI), serum SCr, bilirubin, INR, cold ischemia time, warm ischemia time, MELD score and post OLT complications (as listed above) including AKI and requirement for RRT.

### Definition of acute kidney injury (AKI)

For the definition of acute kidney injury SCr criteria of the KDIGO classification were used, representing either the RIFLE or the AKIN classification [8,11]. According to the KDIGO criteria either a SCr increase of 50% from baseline within one week or a SCr increase of 0.3 mg/dl or greater within 48 h is defined as AKI. The 50% increase was originally derived from the RIFLE classification and will therefore be addressed as RIFLE criteria, whereas the 0.3 mg/dl SCr increase was derived from the AKIN classification and will therefore be addressed as AKIN criteria throughout the manuscript. Some patients fulfill both, AKIN and RIFLE, criteria for AKI. These patients were included into a separate group, whereas patients that fulfilled only either for RIFLE or AKIN were classified separately. Patients expiring AKI requiring RRT (acute renal failure (ARF) literally) were grouped in a forth group and were not included in group 1–3.

### Statistical analysis

For continuous variables the standard descriptive statistics (mean, standard deviation, median, quartiles, minimum, maximum) were computed. For categorical variables description was done via counts and percentages. The endpoint AKI was modeled by univariable and multiple binary logistic regression using age, height, weight, body mass index (BMI), SCr, bilirubin, INR, MELD score, warm and cold ischemia time, time on respirator transfusion of RPC and days on the ICU as independent variables. To this end, the AKI-variable was used as binary variable using “No AKI” as reference group and the other groups as event in separate regression models. Furthermore, only independent variables with a *P*-value below 0.05 were used in the multivariable regressions. In additional regression analyses, the candidate independent variables had to be pre OLT variables to reflect the situation when the outcome of AKI has to be predicted before surgery. To analyze survival, survival rates at 28 days, one year and three years were determined and compared with a global and Bonferroni adjusted pairwise logrank tests for the AKI groups. Survival curves with 95% confidence intervals were generated for the Kaplan–Meier estimates for survival. Crude and adjusted hazard ratios with 95% confidence intervals were computed using univariable and multiple proportional hazards regressions. In additional proportional hazard regressions the candidate independent variables had to be pre OLT, analogue to the binary logistic regressions.

All *P*-values below 0.05 were regarded significant, if not stated otherwise. All analyses were performed with the statistical software SAS 9.2 (SAS Institute Inc., Cary, North Carolina, U.S.A.).

## Results

### Patient population

**Figure 1:**
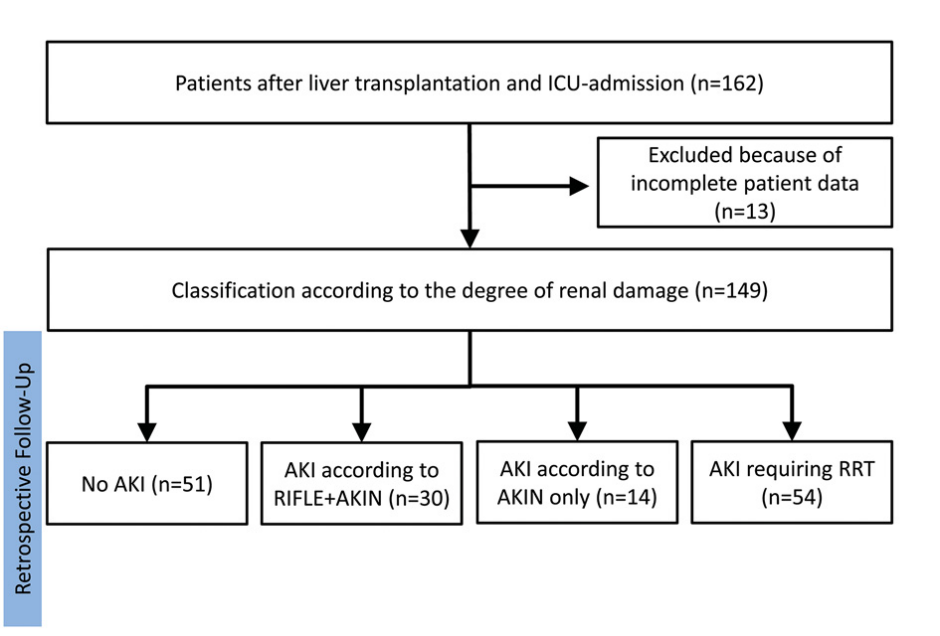
Study design ICU: Intensive care unit, AKI: acute kidney injury, AKIN: Acute Kidney Injury Network, RIFLE: **R**isk **I**njury **F**ailure **L**oss of function **E**nd stage renal disease, RRT: renal replacement therapy

From 1/2004 to 12/2007, we recorded 162 admissions (*m* = 102, *f* = 60) after liver transplantation at our ICU. Of those patients 149 patients (*m* = 94, *f* = 55) fulfilled the inclusion criteria for our study (Figure 1). The mean patient age of all patients was 48.7 ± 12.1 with a BMI of 25.9 ± 4.2 kg/m^2^. The mean MELD at time of transplantation was 17.73 ± 9.01. The preoperative serum creatinine in all patients was 1.3 ± 0.7 mg/dl, mean bilirubin was 6.8 mg/dl (range 0.3–37.4 mg/dl), and mean INR was 1.45 ± 0.50. The underlying diseases for indication of OLT are listed in Table 1. The median ICU stay in all patients was 8 days (range: 1–120 days), and the ventilation time was 88 hours (range: 0–5084 hours).

**Table 1:** Diagnosis leading to transplantation

Diagnosis	Number of patients
Alcoholic related cirrhosis	31
HBV	25
HCV	35
ALF	13
Cryptogenic	15
PSC	13
Autoimmune	6
NET	5
Miscellaneous	19
Total	162

Miscellaneous includes: alpha-1 antitrypsine deficiency (2), Budd-Chiari syndrome (4), Liver angio sarcoma (1), Liver cystadeno carcinoma (1), hemochromatosis (1), primary hepatocellular carcinoma without cirrhosis (1), primary biliary cirrhosis (2) secondary biliary cirrhosis (2), secondary sclerozing cholangitis (1), Wilson’s disease (3).

Abbreviations: ALF, acute liver failure; HBV, hepatitis B virus; HCV, hepatitis C virus; NET, neuroendocrine tumor; PSC, primary sclerozing cholangitis.

### Incidence of AKI according to the RIFLE and AKIN criteria and requirement for RRT

Patients were classified according to their degree of renal damage. Following OLT 30 (20.1%) patients developed AKI according to the RIFLE and AKIN criteria together. The number of patients who developed AKI according only to the AKIN criteria was 14 (9.4%), whereas no patient developed AKI according to the RIFLE criteria only. The incidence of patients who developed AKI requiring RRT (ARF) was 36.2% (*n* = 54), with a total incidence for AKI of 65.7%. The demographics of pre- and intra OLT factors and post OLT complications of the patients according to their AKI group are shown in Table 2.

**Table 2:** Comparison of pre and intraoperative factors and postoperative complications according to the AKI group

	No AKI	AKI no RRT (AKIN+RIFLE)	AKI no RRT (AKIN only)	AKI + RRT (AKIN+RIFLE)	*P*-value
**Number**	**51 (34.2%)**	**30 (20.1%)**	**14 (9.4%)**	**54 (36.2%)**	
Male	29 (56.7%)	22 (73.3%)	9 (64.3%)	34 (63.0%)	
Age (years)	51 ± 11	50 ± 12	48 ± 14	46 ± 13	
Weight (kg)	75 ± 12	80 ± 16	78 ± 13	79 ± 18	
Height (cm)	172 ± 8	173 ± 8	175 ± 12	173 ±10	
BMI (kg/m^2^)	25 ± 3	27 ± 4	26 ± 4	26 ± 5	
MELD	13.4 ± 6.6	15.2 ± 6.2	17.8 ± 6.9*	23.2 ± 10.1‡	*0.05 vs. No AKI ‡ <0.01 vs. No AKI
SCr pre (mg/dl)	1.08 ± 0.37	1.10 ± 0.31	1.21 ± 0.63	1.65 ± 1.01‡	‡ <0.01 vs. No AKI
Bilirubin pre (mg/dl)	4.3 ± 7.0	5.1 ± 5.7	7.2 ± 8.7	10.0 ± 10.0‡	‡ <0.01 vs. No AKI
INR	1.35 ± 0.51	1.37 ± 0.39	1.49 ± 0.51	1.59 ± 0.52‡	‡ <0.03 vs. No AKI
ICU stay (days)	5.4 ± 2.7	8.0 ± 5.01†	10.8 ± 12.4*	21.7 ± 21.4‡	† 0.01 vs. No AKI * 0.04 vs. No AKI ‡ <0.01 vs. No AKI
Ventilation time (hours)	87 ± 227	115 ± 232	154 ± 220	736 ± 846‡	‡ <0.01 vs. No AKI
CIT (min)	409 ± 185	418 ± 212	452 ± 201	411 ± 172	
WIT (min)	33 ± 8	37 ± 11	39 ± 19	74 ± 214	
Tranfusion (units of RPC)	0.8 ± 1.7	2.1 ± 3.9	1.5 ± 2.5	5.9 ± 6.6‡	‡ <0.01 vs. No AKI

AKI: acute kidney injury

AKIN: Acute Kidney Injury Network

RIFLE: **R**isk **I**njury **F**ailure **L**oss of function **E**nd stage renal disease

RRT: renal replacement therapy

BMI: body mass index

CIT: cold ischemia time; WIT: warm ischemia time

RPC: Red packed cells

Pre: preoperative

Values are shown as the mean ± standard deviation.

### Uni- and multivariate analysis of pre and intra OLT factors and post OLT complications for development of AKI

Pre and intraoperative factors and postoperative complications were evaluated for their impact on development of AKI (Table 3). The univariate analysis of the pre and intra OLT factors showed that there was no association with the development of AKI according to the AKIN **and** RIFLE criteria, whereas a higher MELD score was associated with an increased risk for AKI according to the AKIN criteria only. The preoperative factors MELD, SCr, Bilirubin and INR were highly associated with an increased risk for developing AKI requiring RRT in the univariate analysis (Table 3).

**Table 3:** Univariate analysis of pre and intraoperative factors and postoperative complications for the development of AKI

	AKI (AKIN+RIFLE)			AKI (AKIN only)			AKI (RRT)		
	***P*-value**	**OR**	**95% CI**	***P*-value**	**OR**	**95% CI**	***P*-value**	**OR**	**95% CI**
**Pre and intra OLT factor**									
Gender	0.14	2.09	0.78–5.56	0.62	1.37	0.40–4.65	0.52	1.29	0.59–2.82
Age	0.81	1.00	0.96–1.04	0.39	0.98	0.93–1.03	0.08	0.97	0.94–1.00
Weight (kg)	0.10	1.03	0.99–1.06	0.40	1.02	0.97–1.07	0.22	1.02	0.99–1.04
Height (cm)	0.64	1.01	0.96–1.07	0.37	1.03	0.96–1.10	0.60	1.01	0.97–1.05
BMI (kg/m^2^)	0.12	1.10	0.98–1.25	0.71	1.03	0.87–1.23	0.29	1.05	0.96–1.16
MELD	0.26	1.04	0.97–1.12	0.05	1.09	1.00–1.19	<0.01	1.15	1.08–1.22
SCr pre (mg/dl)	0.78	1.21	0.33–4.38	0.34	1.82	0.54–6.16	<0.01	5.23	1.96–14.02
Bilirubin pre (mg/dl)	0.63	1.02	0.95–1.09	0.21	1.05	0.98–1.12	<0.01	1.09	1.03–1.16
INR	0.85	1.10	0.42–2.86	0.39	1.57	0.56–4.41	0.03	3.15	1.13–8.73
CIT (min)	0.84	1.00	1.00–1.00	0.46	1.00	1.00–1.00	0.94	1.00	1.00–1.00
WIT (min)	0.17	1.03	0.99–1.08	0.16	1.04	0.99–1.09	0.06	1.04	1.00–1.08
**Post OLT complications**									
ICU stay (days)	0.01	1.22	1.05–1.42	0.04	1.16	1.00–1.35	<0.01	1.32	1.16–1.50
Ventilation time (h)	0.59	1.00	1.00–1.00	0.35	1.00	1.00–1.00	<0.01	1.01	1.00–1.01
Tranfusion (units of RPC)	0.08	1.22	0.98–1.51	0.23	1.19	0.90–1.57	<0.01	1.76	1.36–2.27

AKI: acute kidney injury

AKIN: Acute Kidney Injury Network

RIFLE: **R**isk **I**njury **F**ailure **L**oss of function **E**nd stage renal disease

RRT: renal replacement therapy

BMI: body mass index

CIT: cold ischemia time; WIT: warm ischemia time

Pre: preoperative

For the post OLT complications, length of ICU stay was associated with the development of AKI regardless of the definition used or the requirement of RRT in the univariate analysis: Patients with a longer ventilation time and a higher amount of RPC transfusion were more likely to have AKI requiring RRT, whereas these parameters were not associated with milder forms of AKI (Table 3).

Multivariate logistic regression was performed using all factors and complications that were found to be significant (*P* < 0.05) in the univariate analysis. MELD score was not included in the multivariate analysis, since it is dependent on creatinine, INR and bilirubin (see ‘Patients and methods’ section). In the multivariate analysis for preoperative factors postoperative complications were not included, because postoperative complications are a consequence of the pre- and intraoperative factors and therefore also dependent on the pre and intra OLT factors. Preoperative SCr was the only factor that was associated with an increased risk for the development of AKI requiring RRT (Table 4). In the analysis of the postoperative complications, length of ICU stay was associated with the development of AKI according to the AKIN criteria only and AKI requiring RRT. Patients with a longer ventilation time and a higher amount of RPC transfusion were also more likely to have AKI requiring RRT in the multivariate analysis (Table 4).

**Table 4:** Multivariate analysis of preoperative factors and postoperative complications for the development of AKI

	AKI (AKIN+RIFLE)			AKI (AKIN only)			AKI (RRT)		
	***P*-value**	**OR**	**95% CI**	***P*-value**	**OR**	**95% CI**	***P*-value**	**OR**	**95% CI**
**Pre OLT factors**									
SCr pre (mg/dl)	n.s.			n.s.			0.01	3.71	1.32–10.42
Bilirubin pre (mg/dl)	n.s.			n.s.			0.18	0.93	0.81–1.07
INR	n.s.			n.s.			0.73	0.11	0.01–1.14
**Post OLT complications**									
ICU stay (days)	0.05	1.16	1.00–1.35	0.01	1.22	1.05–1.42	0.20	1.09	0.96–1.23
Ventilation time (h)	n.s.			n.s.			<0.01	1.00	1.00–1.01
Tranfusion (units of RPC)	n.s.			n.s.			0.03	1.49	1.05–2.11

AKI: acute kidney injury

AKIN: Acute Kidney Injury Network

RIFLE: **R**isk **I**njury **F**ailure **L**oss of function **E**nd stage renal disease

RRT: renal replacement therapy

BMI: body mass index

CIT: cold ischemia time; WIT: warm ischemia time

Pre: preoperative

### Patient survival

**Figure 2:**
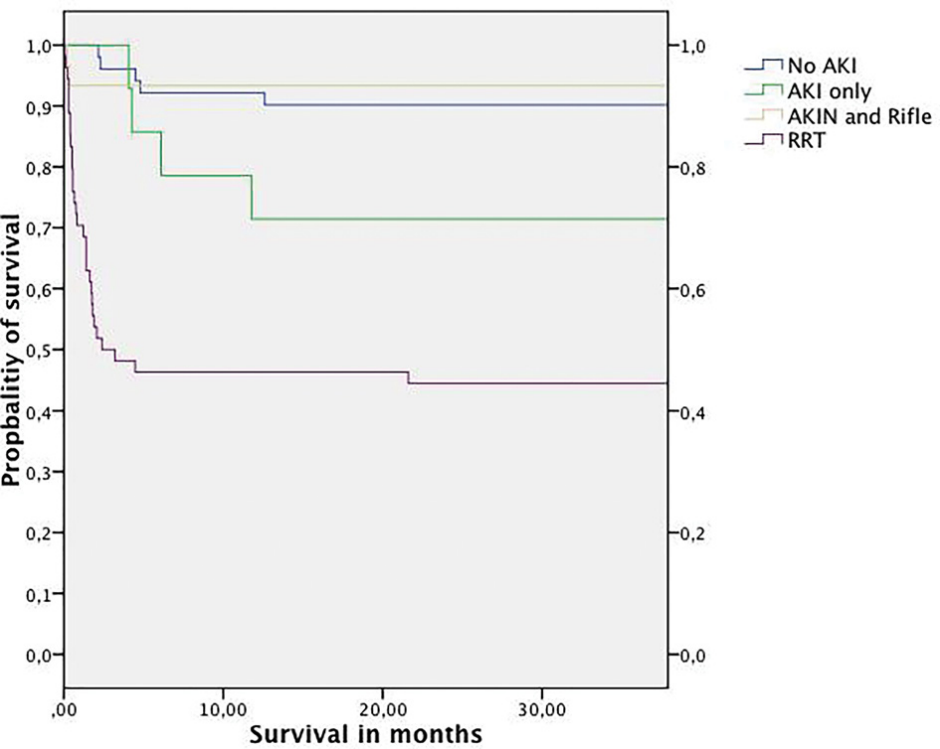
Kaplan–Meier survival curves for the different degree of renal injury Survival was dramatically reduced in patients experiencing acute kidney injury requiring renal replacement therapy (RRT, *N* = 54) compared with patient without acute kidney injury (No AKI, *N* = 51), patients with acute kidney injury according to the AKIN criteria (AKI only, *N* = 14) and patients with acute kidney injury according to the AKIN and RIFLE criteria (AKIN and RIFLE, *N* = 30). There was no patient, who developed acute kidney injury according to the RIFLE criteria only (AKIN: acute kidney injury network, AKI: acute kidney injury, RRT: renal replacement treatment).

Survival rates for patients after OLT at 28 days, one year and three years post OLT are illustrated in Table 5 and Figure 1 along with the impact of the degree of renal injury. The overall patient survival of all patients was 87.9% for 28 days 73.8% for one year and 72.5% for three years. Short-term (28 days) and long-term (one year and three year) survival was dramatically reduced by AKI requiring RRT, whereas AKI without RRT had no impact on survival (Figure 2 and Table 5). Univariate Kaplan–Meier analysis was initially carried out to analyze the influence of various factors (listed in the ‘Methods’ section) on survival and those variables found to be of significance were entered into the multivariate Cox regression model. The results are illustrated in Table 6. Patients expiring AKI that required RRT had a significant higher risk to die both in the analysis for short- and long-term outcome (Table 5 and Figure 2). Transfusion was also associated with a higher risk of death, but this association vanished in the long-term outcome analysis at three years.

**Table 5:** Patient survival

Survival	Median (days)	95% CI	28 d (%)	1 year (%)	3 year (%)	*P* vs. No AKI
No AKI	1561	1380–1742	100	92.2	90.2	
AKI (AKIN+RIFLE)	1179	1281–1706	93.3	93.3	93.3	1.000
AKI (AKIN only)	1494	744–1613	100	71.4	71.4	0.068
AKI (RRT)	86	0*–310	70.4	46.4	44.4	<0.001

Log rank test for analysis of the effect of the degree of renal injury on patient survival

AKI: acute kidney injury

AKIN: Acute Kidney Injury Network

RIFLE: **R**isk **I**njury **F**ailure **L**oss of function **E**nd stage renal disease

RRT: renal replacement therapy

CI: confidence interval

*Lower border of the confidence interval was set to 0 for logical reasons.

**Table 6:** Cox regression multivariate analysis of the effect of different factors on patient survival

	28 d (%)			1 year (%)			3 year (%)		
	*P*-value	HR	95% CI	*P*-value	HR	95% CI	*P*-value	HR	95% CI
Transfusion	< 0.001	1.08	1.04-1.12	0.049	1.05	1.00–1.11	0.115		
AKI (RRT)	*	*	*	<0.001	10.63	3.48–32.45	<0.001	8.46	3.06–23.40
Ventilation time	–			0.646			0.295		
ICU stay	–			0.260			–		

HR: Hazard ratio

AKI: acute kidney injury

AKIN: Acute Kidney Injury Network

RIFLE: **R**isk **I**njury **F**ailure **L**oss of function **E**nd stage renal disease

RRT: renal replacement therapy

CI: confidence interval

* Due to the fact that there were no death in the No AKI group a hazard ratio could not be calculated. The logrank test for 28 days survival of AKI (RRT) vs. no AKI was highly significant (logrank *P* < 0.001).

## Discussion

The true incidence of postoperative AKI after OLT is not known due to different patient selections, different methods and definitions of AKI. However, the rate seems to be very high [10,18,19]. Caebezuelo et al. [20] evaluated the incidence of AKI in liver transplant patients. In their study, the incidence of AKI was reported with 50%. Bilbao et al [19] showed in their series comparable incidence of AKI after OLT (51.1%). In our study the total incidence of AKI was slightly higher (65.8%), probably based on the fact that we used the strict AKIN criteria for the definition of AKI. In a very recent study Hilmi et al. reported that the incidence of AKI following OLT was 52% [21]. The incidence is comparable with the study of Bilbao and our data. Looking at more sever forms of AKI our data are in contrast with the study of Gonwa et al. [1], who reported a rate of RRT dependent AKI of 8.3% among patients transplanted between 1985 and 1995, and 12.5% among patients transplanted between 1995 and 1999, whereas the rate in our study was 36.2%. O’Riordan et al. [22] evaluated OLT recipients with AKI defined by RIFLE criteria. In this series the whole number of AKI was 36.8%, and the number of RRT dependent kidney failure was 25.7%, which is closer to our data. The reason for the different incidence of more severe kidney injury may be that O’Riordans and our patients were transplanted later. Unfortunately, the total quality of donor organs has seen a continuous deterioration over the last decades in the Eurotransplant (ET) region [23]. Furthermore, the quality of the transplanted organs assessed by the donor-risk-index (DRI) [24] is significantly worse in the ET region compared with the U.S.A. [25]. This is aggravated by the fact that an increasing number of institutions, including ours had to use lower quality graft in selected patients to overcome the organ shortage [26]. This may lead to a higher incidence of more severe AKI caused by an initially poorer graft function. The second issue that may cause the increased rate of severe AKI in our study may be related to the MELD-based allocation system. Since 12/2006 the allocation of organs in Germany is based on the MELD score [23]. Caused by this allocation modus the MELD at time of transplantation increased from 25 in 1/2007 to 34 in 12/2010. As SCr has an impact on the MELD score (see ‘Methods’ section), this implies that liver transplantation is performed in patients with a higher degree of kidney damage, which would predispose to a higher incidence of more severe forms of AKI (see below). However, in agreement with all the mentioned studies we confirm that OLT is a high-risk procedure leading to significant renal damage with a high amount of patients progressing to renal failure requiring RRT.

Reasons for renal damage after OLT are episodes of hypotension or the use of nephrotoxic antibiotics like aminoglyocosides. These reasons are well documented and studied [22,27,28,29]. Other reasons for renal damage were shown by Hilmi et al. including a body weight > 100 kg, female gender, Child-Pugh score and pre-exsiting diabetes. The fact that increased body weight was associated with increased postoperative kidney failure has already been demonstrated in patients undergoing cardiovascular surgery [23]. Utsimi et al. [24] showed that pre-existing diabetes and MELD score ≥ 20 were risk factors for renal damage after OLT. In our study, a preoperative higher MELD score was also associated with postoperative RRT dependent AKI. However, in contrast with Hilmi et al., body weight was not a risk factor for postoperative AKI, probably because we had fewer patients with a body weight of > 100 kg in our study. There are some reports indicating that pre-OLT renal dysfunction is associated with post-OLT dialysis [30,31]. We also found that patients with a pre-OLT kidney dysfunction assessed by SCr developed significantly more AKI requiring RRT, but there was no correlation with milder forms of AKI. This is in line with the study of O’Riordan et al. [22], who found that SCr was associated with severe AKI requiring RRT, but not with milder forms of AKI. Bilbao and colleagues [19] reported that an increased Scr (> 1.5 mg/dl) was highly associated with severe AKI (serum creatinine > 3 mg/dl requiring RRT). It is interesting that pre OLT SCr only predicted more severe forms of AKI and did not predict AKI in general. This is probably related to the fact that SCr as a marker for AKI does not distinguish between functional and structural decline in renal function. The functional decline in renal function commonly encountered in critical care patients (e.g. by volume shifts) may therefore present as milder forms of AKI. This functional decline in renal function does occur regardless of the underlying renal damage before OLT, whereas structural damage of AKI, presented by AKI requiring RRT, is associated with the underlying renal damage before OLT. In summary, pre OLT SCr is the most important risk factor for the development of AKI requiring RRT.

Most studies evaluating AKI in non OLT patients showed a higher mortality rate in patients with AKI and patients with dialysis dependent AKI [32–35]. We used in our study the KDIGO definition based on the RIFLE and AKIN criteria to reflect the advance in the definition of AKI, but also to compare the present study to other studies, where RIFLE and AKIN criteria were used [22,36,37]. Additionally, we could compare both definitions head to head. We supposed that the AKIN criteria would be more relevant for OLT patients, since these criteria appear to be more sensitive to milder forms of renal damage. It is still common practice to assess kidney function with serum creatinine level. Patients with ESLD has lower serum creatinine levels for two reasons:

(i) Less muscle mass and muscle activity and (ii) increased extracellular space, why creatinine appears normal, although it pathological. For that reason, this tight AKI definition appears beneficial for cirrhotic patients.

To our surprise AKI assessed by the AKIN or RIFLE criteria had the same survival rate as patients without AKI and there was no relevant difference between both definitions. However, patients with AKI requiring RRT had significantly reduced survival as opposed to the other groups. This reduced survival rate was still present even at 3 years. There are several attempts to explain why patients with dialysis dependent renal failure have increased mortality, even 3 years after transplantation. It seems that the balance between pro- and anti-inflammation is tilt. Post-ischemic inflammation contributes to tissue damage in AKI although inflammatory processes are involved in kidney repair as well. The outer medulla displays the highest vulnerability toward ischemia-induced damage. Inflammatory processes in this compartment partly result from endothelial up-regulation of certain cell adhesion molecules such as intercellular adhesion molecule 1 and 2 (ICAM-1 and -2), CD99 and proteins of the junctional adhesion molecule family (JAM) [38]. This may be followed by prolonged antiinflammation that ends in immunoparalysis with a risk of superinfection. Additionally, poor liver function may contribute to the high mortality.

O’Riordan et al. [22] evaluated also the 30-day and 1-year survival in OLT patients with AKI. They showed that patients experiencing milder forms of AKI assessed by the RIFLE criteria had no loss in survival, whereas patients experiencing more severe forms of AKI (RRT requirement) had a significant decrease in survival after 30 days and one year. One-year survival in the present study in patients with AKI requiring RRT was very similar to our results (47.5% vs. 46.4%). Another study published by Barri et al. [39] evaluated AKI in OLT patients defined by SCr rise from baseline. In contrast with our study and the study of O’Riordan they showed that even patients with mild AKI (SCr rise of > 0.5 mg/dl) had reduced survival. In a more recent study, where the three different degrees of severity proposed by AKIN were used, a significant reduction in survival was shown, even in patients with stage one AKI after OLT. More severe degrees of renal damage (AKIN 2-3) showed worse outcome [40]. A more recent report dealing with the question, whether milder forms of AKI are associated with poor outcome, was published by Romano and colleagues. In the present study, the authors were not able to demonstrate an association between milder forms of AKI and mortality, whereas more severe forms of AKI with RRT requirement were associated with a worse outcome [41]. To clarify the question whether mild forms of renal damage have an impact on patient survival after OLT, more studies are needed. However, it is unquestionable that severe forms of AKI, especially those that require RRT, have a huge impact on patient survival indicating a clinical distinction between milder and more severe forms of AKI. The dilemma is that we do not have good biomarkers to assess whether a patient will progress to AKI requiring RRT or not. But since this discrimination is so critical, we really need these biomarkers. This could then guide us to determine the optimal time for the start of RRT. Fortunately, some biomarkers are under evaluation that might be of use in this situation even though they were not evaluated in patients after OLT yet [42].

Because preoperative kidney function plays a crucial role for postoperative kidney failure, the question arises, if those patients would benefit from simultaneous liver–kidney transplantation. Several consensus conferences discussed this issue in order to develop an appropriate algorithm for this setting [43–45]. Brennan et al. [46] compared the outcome of patients with preoperative renal dysfunction, who underwent liver transplantation alone or simultaneous liver–kidney transplantation. One-year survival in patients with renal dysfunction and liver transplantation alone had worse outcome compared with patients who received combined liver–kidney transplantation (79.6% vs. 91.2%, *P* = 0.05). However, patients receiving only liver transplantation were sicker than patients receiving combined transplant (MELD: 37.9 vs. 32.7, *P* = 0.0004). The authors concluded that their own data do not provide evidence to recommend combined liver–kidney transplantation in patients suffering from liver and kidney failure. Another option for these patients would be kidney transplantation 3–6 months after liver transplantation. This would allow time to appreciate the regeneration of the kidney after liver transplantation and decide whether kidney transplantation is still necessary.

In conclusion, pre OLT renal dysfunction assessed by SCr remains the most important risk factor predicting severe forms of AKI that require RRT. However, pre OLT renal dysfunction was not able to predict milder forms of AKI. Our data indicate that AKI requiring RRT is of major clinical significance, affecting ventilation time, requirement of blood transfusion and most importantly patient survival.

## Abbreviations

ADQI: Acute Dialysis Qualitative Initiative
AKI: acute kidney injury
AKIN: Acute Kidney Injury Network
aPTT: activated partial thromboplastin time
ARF: acute renal failure
BMI: body mass index
CNI: calcineurin-inhibitor
CVVHD: continuous veno-venous hemodialysis
DRI: donor risk index
ECD: extended criteria donors
ESLD: endstage-liver-disease
ICAM: intercellular adhesion molecule
ICU: intensive care unit
JAM: junctional adhesion molecule family
KDIGO: Kidney Disease Improving Global Outcome
MELD: model end-stage liver disease
OLT: orthotopic liver transplantion
RPC: red packed cells
RRT: renal replacement therapy
SCr: serum creatinine

## References

[1] Gonwa T.A., Mai M.L., Melton L.B., Hays S.R., Goldstein R.M., Levy M.F. et al. (2001) Renal replacement therapy and orthotopic liver transplantation: the role of continuous veno-venous hemodialysis. Transplantation 71, 1424–1428 10.1097/00007890-200105270-00012 11391230

[2] Barri Y.M., Sanchez E.Q., Jennings L.W., Melton L.B., Hays S., Levy M.F. et al. (2009) Acute kidney injury following liver transplantation: definition and outcome. Liver Transpl. 15, 475–483 10.1002/lt.21682 19399734

[3] Saner F.H., Cicinnati V.R., Sotiropoulos G. and Beckebaum S. (2012) Strategies to prevent or reduce acute and chronic kidney injury in liver transplantation. Liver Int. 32, 179–188 10.1111/j.1478-3231.2011.02563.x 21745304

[4] Saner F.H., Treckmann J.W., Geis A., Losch C., Witzke O., Canbay A. et al. (2012) Efficacy and safety of regional citrate anticoagulation in liver transplant patients requiring post-operative renal replacement therapy. Nephrol. Dial. Transplant. 27, 1651–1657 10.1093/ndt/gfr510 22049184

[5] Dharancy S., Iannelli A., Hulin A., Declerck N., Schneck A.S., Mathurin P. et al. (2009) Mycophenolate mofetil monotherapy for severe side effects of calcineurin inhibitors following liver transplantation. Am. J. Transplant. 9, 610–613 10.1111/j.1600-6143.2008.02529.x 19260838

[6] Gonwa T.A., Mai M.L., Melton L.B., Hays S.R., Goldstein R.M., Levy M.F. et al. (2001) End-stage renal disease (ESRD) after orthotopic liver transplantation (OLTX) using calcineurin-based immunotherapy: risk of development and treatment. Transplantation 72, 1934–1939 10.1097/00007890-200112270-00012 11773892

[7] Mehta R.L. and Chertow G.M. (2003) Acute renal failure definitions and classification: time for change? J. Am. Soc. Nephrol. 14, 2178–2187 10.1097/01.ASN.0000079042.13465.1A 12874474

[8] Mehta R.L., Kellum J.A., Shah S.V., Molitoris B.A., Ronco C., Warnock D.G. et al. (2007) Acute Kidney Injury Network: report of an initiative to improve outcomes in acute kidney injury. Crit. Care 11, R31 10.1186/cc5713 17331245 PMC2206446

[9] Hobson C.E., Yavas S., Segal M.S., Schold J.D., Tribble C.G., Layon A.J. et al. (2009) Acute kidney injury is associated with increased long-term mortality after cardiothoracic surgery. Circulation 119, 2444–2453 10.1161/CIRCULATIONAHA.108.800011 19398670

[10] McCauley J., Van Thiel D.H., Starzl T.E. and Puschett J.B. (1990) Acute and chronic renal failure in liver transplantation. Nephron 55, 121–128 10.1159/000185938 2362625 PMC2957102

[11] Bellomo R., Ronco C., Kellum J.A., Mehta R.L. and Palevsky P. (2004) Acute renal failure - definition, outcome measures, animal models, fluid therapy and information technology needs: the Second International Consensus Conference of the Acute Dialysis Quality Initiative (ADQI) Group. Crit. Care 8, R204–R212 10.1186/cc2872 15312219 PMC522841

[12] Weisbord S.D., Chen H., Stone R.A., Kip K.E., Fine M.J., Saul M.I. et al. (2006) Associations of increases in serum creatinine with mortality and length of hospital stay after coronary angiography. J. Am. Soc. Nephrol. 17, 2871–2877 10.1681/ASN.2006030301 16928802

[13] Lassnigg A., Schmid E.R., Hiesmayr M., Falk C., Druml W., Bauer P. et al. (2008) Impact of minimal increases in serum creatinine on outcome in patients after cardiothoracic surgery: do we have to revise current definitions of acute renal failure. Crit. Care Med. 36, 1129–1137 10.1097/CCM.0b013e318169181a 18379238

[14] Saner F.H., Cicinnati V.R., Sotiropoulos G. and Beckebaum S. (2012) Strategies to prevent or reduce acute and chronic kidney injury in liver transplantation. Liver Int. 32, 179–185 21745304 10.1111/j.1478-3231.2011.02563.x

[15] (2012) KDIGO Clinical Practice Guideline for Acute Kidney Injury. Kidney Int. Suppl. 2, 1–138

[16] Saner F.H., Olde Damink S.W., Pavlakovic G., van den Broek M.A., Rath P.M., Sotiropoulos G.C. et al. (2008) Pulmonary and blood stream infections in adult living donor and cadaveric liver transplant patients. Transplantation 85, 1564–1568 10.1097/TP.0b013e31816f61a6 18551060

[17] Kamath P.S., Wiesner R.H., Malinchoc M., Kremers W., Therneau T.M., Kosberg C.L. et al. (2001) A model to predict survival in patients with end-stage liver disease. Hepatology 33, 464–470 10.1053/jhep.2001.22172 11172350

[18] Rimola A., Gavaler J.S., Schade R.R., el-Lankany S., Starzl T.E. and Van Thiel D.H. (1987) Effects of renal impairment on liver transplantation. Gastroenterology 93, 148–156 10.1016/0016-5085(87)90327-1 3556303

[19] Bilbao I., Charco R., Balsells J., Lazaro J.L., Hidalgo E., Llopart L. et al. (1998) Risk factors for acute renal failure requiring dialysis after liver transplantation. Clin. Transplant. 12, 123–129 9575400

[20] Cabezuelo J.B., Ramirez P., Rios A., Acosta F., Torres D., Sansano T. et al. (2006) Risk factors of acute renal failure after liver transplantation. Kidney Int. 69, 1073–1080 10.1038/sj.ki.5000216 16528257

[21] Hilmi I.A., Damian D., Al-Khafaji A., Planinsic R., Boucek C., Sakai T. et al. (2015) Acute kidney injury following orthotopic liver transplantation: incidence, risk factors, and effects on patient and graft outcomes. Br. J. Anaesth. 10.1093/bja/aeu556 25673576

[22] O’Riordan A., Wong V., McQuillan R., McCormick P.A., Hegarty J.E. and Watson A.J. (2007) Acute renal disease, as defined by the RIFLE criteria, post-liver transplantation. Am. J. Transplant. 7, 168–176 10.1111/j.1600-6143.2006.01602.x 17109735

[23] Schlitt H.J., Loss M., Scherer M.N., Becker T., Jauch K.W., Nashan B. et al. (2011) Current developments in liver transplantation in Germany: MELD-based organ allocation and incentives for transplant centres. Z. Gastroenterol. 49, 30–38 10.1055/s-0029-1245946 21225535

[24] Feng S., Goodrich N.P., Bragg-Gresham J.L., Dykstra D.M., Punch J.D., DebRoy M.A. et al. (2006) Characteristics associated with liver graft failure: the concept of a donor risk index. Am. J. Transplant. 6, 783–790 10.1111/j.1600-6143.2006.01242.x 16539636

[25] Braat A.E., Blok J.J., Kooreman N.G., Dubbeld J., Putter H., Rahmel A.O. et al. (2009) Preliminary Results of a Study for Liver Donor Quality within the Eurotransplant Database: Striking Differences with the Unos Liver Donor Quality. Transpl. Int. 22, 92–92

[26] Sotiropoulos G.C., Paul A., Gerling T., Molmenti E.P., Nadalin S., Napieralski B.P. et al. (2006) Liver transplantation with “rescue organ offers” within the eurotransplant area: a 2-year report from the University Hospital Essen. Transplantation 82, 304–309 10.1097/01.tp.0000229447.37333.ed 16906024

[27] Moore R.D., Smith C.R., Lipsky J.J., Mellits E.D. and Lietman P.S. (1984) Risk factors for nephrotoxicity in patients treated with aminoglycosides. Ann. Intern. Med. 100, 352–357 10.7326/0003-4819-100-3-352 6364908

[28] Contreras G., Garces G., Quartin A.A., Cely C., LaGatta M.A., Barreto G.A. et al. (2002) An epidemiologic study of early renal replacement therapy after orthotopic liver transplantation. J. Am. Soc. Nephrol. 13, 228–233 11752042 10.1681/ASN.V131228

[29] Park Y., Hirose R., Dang K., Xu F., Behrends M., Tan V. et al. (2008) Increased severity of renal ischemia-reperfusion injury with venous clamping compared to arterial clamping in a rat model. Surgery 143, 243–251 10.1016/j.surg.2007.07.041 18242341

[30] Lebron Gallardo M., Herrera Gutierrez M.E., Seller Perez G., Curiel Balsera E., Fernandez Ortega J.F. and Quesada Garcia G. (2004) Risk factors for renal dysfunction in the postoperative course of liver transplant. Liver Transpl. 10, 1379–1385 10.1002/lt.20215 15497160

[31] Sanchez E.Q., Gonwa T.A., Levy M.F., Goldstein R.M., Mai M.L., Hays S.R. et al. (2004) Preoperative and perioperative predictors of the need for renal replacement therapy after orthotopic liver transplantation. Transplantation 78, 1048–1054 10.1097/01.TP.0000137176.95730.5B 15480173

[32] Abosaif N.Y., Tolba Y.A., Heap M., Russell J. and El Nahas A.M. (2005) The outcome of acute renal failure in the intensive care unit according to RIFLE: model application, sensitivity, and predictability. Am. J. Kidney Dis. 46, 1038–1048 10.1053/j.ajkd.2005.08.033 16310569

[33] Bell M., Liljestam E., Granath F., Fryckstedt J., Ekbom A. and Martling C.R. (2005) Optimal follow-up time after continuous renal replacement therapy in actual renal failure patients stratified with the RIFLE criteria. Nephrol. Dialysis Transplant. 20, 354–360 10.1093/ndt/gfh581 15598666

[34] Hoste E.A., Clermont G., Kersten A., Venkataraman R., Angus D.C., De Bacquer D. et al. (2006) RIFLE criteria for acute kidney injury are associated with hospital mortality in critically ill patients: a cohort analysis. Crit. Care 10, R73 10.1186/cc4915 16696865 PMC1550961

[35] Bihorac A., Yavas S., Subbiah S., Hobson C.E., Schold J.D., Gabrielli A. et al. (2009) Long-term risk of mortality and acute kidney injury during hospitalization after major surgery. Ann. Surg. 249, 851–858 10.1097/SLA.0b013e3181a40a0b 19387314

[36] Lima E.Q., Zanetta D.M., Castro I., Massarollo P.C., Mies S., Machado M.M. et al. (2003) Risk factors for development of acute renal failure after liver transplantation. Ren. Fail. 25, 553–560 10.1081/JDI-120022546 12911159

[37] Che M., Li Y., Liang X., Xie B., Xue S., Qian J. et al. (2011) Prevalence of acute kidney injury following cardiac surgery and related risk factors in Chinese patients. Nephron Clin. Pract. 117, c305–c311 10.1159/000321171 20861652

[38] Scheiermann C., Colom B., Meda P., Patel N.S., Voisin M.B., Marrelli A. et al. (2009) Junctional adhesion molecule-C mediates leukocyte infiltration in response to ischemia reperfusion injury. Arterioscler. Thromb. Vasc. Biol. 29, 1509–1515 10.1161/ATVBAHA.109.187559 19574560 PMC2746810

[39] Barri Y.M., Sanchez E.Q., Jennings L.W., Melton L.B., Hays S., Levy M.F. et al. (2009) Acute kidney injury following liver transplantation: definition and outcome. Liver Transpl. 15, 475–483 10.1002/lt.21682 19399734

[40] Zhu M., Li Y., Xia Q., Wang S., Qiu Y., Che M. et al. (2010) Strong impact of acute kidney injury on survival after liver transplantation. Transplant. Proc. 42, 3634–3638 10.1016/j.transproceed.2010.08.059 21094830

[41] Romano T.G., Schmidtbauer I., Silva F.M., Pompilio C.E., D’Albuquerque L.A. and Macedo E. (2013) Role of MELD Score and Serum Creatinine as Prognostic Tools for the Development of Acute Kidney Injury after Liver Transplantation. PLoS One 8, e64089 10.1371/journal.pone.0064089 23717537 PMC3662723

[42] Kashani K., Al-Khafaji A., Ardiles T., Artigas A., Bagshaw S.M., Bell M. et al. (2013) Discovery and validation of cell cycle arrest biomarkers in human acute kidney injury. Crit. Care 17, R25 10.1186/cc12503 23388612 PMC4057242

[43] Davis C.L. (2008) Controversies in combined liver-kidney transplantation: indications and outcomes. Transplant. Rev. 22, 82–88 10.1016/j.trre.2007.03.006 18631861

[44] Eason J.D., Gonwa T.A., Davis C.L., Sung R.S., Gerber D. and Bloom R.D. (2008) Proceedings of Consensus Conference on Simultaneous Liver Kidney Transplantation (SLK). Am. J. Transplant. 8, 2243–2251 10.1111/j.1600-6143.2008.02416.x 18808402

[45] Nadim M.K., Davis C.L., Sung R., Kellum J.A. and Genyk Y.S. (2012) Simultaneous liver-kidney transplantation: a survey of US transplant centers. Am. J. Transplant. 12, 3119–3127 10.1111/j.1600-6143.2012.04176.x 22759208

[46] Brennan T.V., Lunsford K.E., Vagefi P.A., Bostrom A., Ma M. and Feng S. (2015) Renal outcomes of simultaneous liver-kidney transplantation compared to liver transplant alone for candidates with renal dysfunction. Clin. Transplant. 29, 34–43 10.1111/ctr.12479 25328090 PMC4800976

